# Podiatrists' Reflections on Content and Delivery of Their Pre‐Registration Podiatry Programme at a Regional University in New South Wales, Australia: A Survey of Graduates

**DOI:** 10.1002/jfa2.70053

**Published:** 2025-08-02

**Authors:** Wen Ting Hazel Chua, Antoni Fellas, Andrea Coda, Fiona Hawke

**Affiliations:** ^1^ School of Health Sciences The University of Newcastle Newcastle Australia; ^2^ Equity in Health and Wellbeing Research Program Hunter Medical Research Institute Newcastle Australia

**Keywords:** content, delivery, education, podiatrists, preferences, undergraduate

## Abstract

**Introduction:**

Understanding podiatrists' perceptions of their undergraduate education is important to ensure that educational content and delivery meets the needs of the current workforce to inform future planning. This study aims to explore podiatrists' perceptions of their undergraduate podiatry training at the University of Newcastle, Australia, and their preferences regarding educational content and delivery.

**Methods:**

We conducted an online survey of podiatry graduates from the University of Newcastle, Australia. Data were analysed using descriptive statistics and Fisher's exact test to compare responses between groups. Qualitative responses were analysed using inductive content analysis.

**Results:**

A total of 114 podiatrists responded. Nail avulsions, business management and modifying orthoses were perceived as being given insufficient time and focus in undergraduate training, with a higher proportion of private (71%) compared to public (33%) podiatrists reporting business management as lacking (*p* = 0.02). There was strong support for embedding endorsed scheduled medicines training within the programme (80%) and for delivering theoretical content face‐to‐face rather than online. Inductive content analysis revealed four areas to be emphasised in future curricula: modern technologies, biomechanics, wound care and routine podiatric care. Potential strategies to reduce examination stress included mock assessments, changed assessment weighting, reduced exam structure rigidity and reducing assessor bias.

**Conclusions:**

This study provides insights into Australian podiatrists' preferences for pre‐registration curricula. Topics to emphasise in future curricula at the University of Newcastle, Australia, include greater manual skills and business training, modern technologies, biomechanics and routine podiatric care. Our results suggest exercising caution when substituting face‐to‐face with online learning. These findings provide valuable guidance for future curricula in a context of declining student numbers and increasing healthcare demands.

## 1 Introduction

Podiatrists play an important role in the primary care of patients with chronic disease to reduce disability and improve quality of life, as well as decrease long‐term healthcare costs [1]. Given the growing burden of chronic diseases such as diabetes mellitus [2], and the challenges of an ageing society, the demand for podiatrists is strong and increasing [3, 4]. In September 2023, the NSW Ministry of Health in Australia identified podiatry as a ‘Category One’ occupation, being an occupation with a critical national shortage [5].

Despite the high demand for podiatrists, enrolments to study podiatry at universities have declined by 17% across Australia and New Zealand [6] and by 40% in the United Kingdom [4]. Reasons may include perceived job unavailability, lack of awareness and misconception of the profession as well as a relative increase in enrolments for programmess such as physiotherapy [7]. Unlike other skill shortages which can be addressed through migrant worker programmes, the podiatry profession largely depends on locally trained podiatrists, simply because podiatry does not exist in many developing countries. Podiatry is on Australia's skilled migration list; however, podiatry does not exist in the three countries that are the main contributors to Australia's Skilled Migration Programme (India, The Peoples Republic of China and Philippines) [8]. Therefore, countries such as Australia, England and New Zealand are focusing on increasing enrolments in domestic podiatry programmess to address the workforce shortage.

Several studies have identified potential strategies to increase the number of student enrolments in podiatry programmes, such as offering programmes at esteemed universities and emphasising programme content relating to biomechanics, sports and surgery [9]. Programme graduates are a valuable resource for providing ideas about implementing programme curriculum as they can provide feedback that may not be considered by internal stakeholders such as current staff and students [10, 11]. In previous research, podiatrists have suggested that podiatry programmes could include more health promotion education [12], integrate interprofessional practice [13], provide a spiralling content delivery approach with greater paediatric education [14] and include more pain education [15]. However, understanding podiatrists' perceptions of course content across diverse subject areas such as workplace conflict and business management and delivery methods of podiatry programmes, remains limited. With many training programmes moving to online delivery since COVID‐19, considering the support for online delivery from podiatrists is important to guide future planning. Furthermore, although Graham et al. [16] identified barriers to and facilitators of endorsement for scheduled medicines among podiatrists, support for providing this training in pre‐registration podiatry programmes remains unclear. At the time of planning the current research, the University of Newcastle Podiatry Programme was flagged for major revision. This study aimed to survey graduates of the University of Newcastle Podiatry Programme to explore their preferences regarding content and delivery methods for the new undergraduate podiatry programme.

## 2 Methods

### 2.1 Design

This research was conducted as part of Hazel Chua's honours research. We distributed an online survey developed specifically for this study to podiatrists who had graduated from the University of Newcastle. We opted to include only graduates from the University of Newcastle Podiatry Programme because [1] the results were to inform the redevelopment of that program; [2] we expected that by including only graduates from the University of Newcastle and recruiting through our ‘University of Newcastle Podiatry graduates and students’ Facebook group, our response rate would be fairly high, and higher than if we attempted to recruit podiatry graduates nation‐wide; and [3] due to time constraints of the honours programme. This research was approved by the University of Newcastle Human Research Ethics Committee (HREC) (Approval number H‐2023‐0295). Potential respondents were invited to contact the researchers via email for the participant information statement. A consent statement was included in the online landing page, emphasising voluntary participation and the ability to withdraw from the study without consequence. Consent was collected by participants clicking on the ‘I agree’ button.

### 2.2 Participants

Participants were sourced from the private Facebook group ‘University of Newcastle Podiatry Graduates and Students’. The Facebook group is the most commonly used method for the University of Newcastle podiatry staff to contact former graduates. Since 2017, when the group was established, all students at the University of Newcastle Podiatry Programme have been invited to join via an email sent to all students and announcements at programme lectures. Invitations to join the group for podiatrists who graduated before 2017 (the podiatry programme's first cohort graduated in 2009) have also been distributed by word of mouth through professional and personal networks. The group is curated by a senior lecturer (F.H.) within the podiatry programme. Participation in the Facebook group is voluntary and there are no financial incentives for members or for the curator.

As of the end of 2023, when the survey was conducted, the Facebook group comprised approximately 450 members (*n* = 450), including podiatry graduates and students from the University of Newcastle. This figure represents approximately two‐thirds of the total graduate population since the inception of the podiatry programme in 2009, with an estimated 650 graduates (*n* = 650) having completed the programme between 2009 and 2023. Podiatrists who had graduated from the programme but were not currently working as a podiatrist were included. As the Facebook group also contains current podiatry students who have not yet graduated, we emphasised in the Facebook posts that the survey was for graduates only. Additionally, the first question of the survey asked participants if they had obtained a podiatry degree from the University of Newcastle or if they were currently a student. Participants who selected ‘No’ or ‘Currently a student’ were automatically excluded.

### 2.3 Sample Size

An analysis of existing posts within the Facebook group revealed that each post was viewed by approximately 120 members. To satisfy the central limit theorem [17], an estimated one in four people who viewed each post (*n* = 30 people) were anticipated to complete the survey.

### 2.4 Recruitment

Participants were recruited through the Facebook group and Facebook Messenger chat. Posts contained a link to the online survey hosted by the survey platform, QuestionPro (licenced to the University of Newcastle). The survey was available for 10 days, from 21 to 30 August 2023. On day one, a post was delivered in the Facebook group and to all members of the group via the Messenger Chat function inviting podiatry graduates to complete an online survey about their perceptions of their undergraduate training. The survey was also promoted through email and text to the University of Newcastle graduate podiatrists via word of mouth through personal/professional networks. A follow‐up post was made on day 5 and on another on day 8 (no further Messenger Chat messages were delivered). Respondents were invited to share the survey link with nongroup peers who were also podiatry graduates from the University of Newcastle.

### 2.5 Survey Questions

The survey was developed by H.C. and F.H., reviewed by A.C. and A.F., and was pilot tested by three clinical podiatrists who were not University employees. Adjustments were made to survey questions based on feedback. Demographic questions covered year of graduation, work status, primary setting (private or public practice or a mix or both) and endorsement status for prescribing scheduled medicines.

Survey data included the sufficiency of time and focus on podiatry skill development across different clinical and nonclinical (e.g., business management) topics, with responses ranked on a 5‐point Likert scale from ‘By far not enough’ to ‘By far too much’. Participants were also asked to agree or disagree with statements on online learning, the hybrid‐apprenticeship learning model and assessment relevance, ranked on a 5‐point Likert scale from ‘Strongly disagree’ to ‘Strongly agree’. A range of delivery methods was presented for participants to select which of those that would have enhanced their learning, with participant able to select more than one option. At the end of the survey, participants were asked two open‐ended questions: (1) What additional skills or technology would you like to see included in the training for current and future students in podiatry and (2) Can you think of any strategies students or teachers could use to reduce stress in clinical patient‐based exams (without compromising the integrity of the exam)? If yes, please explain.

### 2.6 Statistical Analysis

Analysis of survey data was conducted following good practice recommendations [18]. Data were analysed using IBM SPSS Statistics (Version 29.0) (Chicago, IL, US). Quantitative data were analysed using descriptive statistics, bar charts and pie charts to summarise responses. To explore associations between private or public practice with opinions on sufficiency of time and focus given to certain subjects, we performed Fisher's exact tests with the two‐sided significance level of *p* < 0.05. We collapsed the outcome categories ‘by far not enough’ with ‘not enough’; and ‘by far too much’ with ‘too much’. We also removed neutral respondents and respondents who worked in a mix of private and public practice. To explore whether the year of graduation was associated with podiatrists' preference for delivery of the course online, we used the nonparametric test, Kendall's tau‐b, as data were not normally distributed (Kolmogorov–Smirnov Test *p* < 0.001).

Analysis of qualitative responses was undertaken using inductive content analysis [19]. One author (H.C.) analysed the data and developed categories and subcategories that arose from the data (all checked by F.H.), with a subsample independently analysed by a second author (F.H.). Consensus was reached by discussion. The process of coding, organisation and categorisation was done manually in Microsoft Excel.

## 3 Results

### 3.1 Participants

A total of 114 podiatrists completed the survey. The majority were living in Australia (*n* = 108, 95%), most were currently working as podiatrists (*n* = 109, 96%) and most worked in private practice (*n* = 97, 87%) (Table 1). The year of graduation ranged from 2009 to 2022 (median 2017).

**TABLE 1:** Descriptive characteristics of podiatrists who responded to the online survey (*n* = 114).

Characteristic	*N* (%)
Country of residence	
Australia	108 (95)
Great Britain	1 (1)
Malaysia	1 (1)
New Zealand	1 (1)
Singapore	2 (2)
Vietnam	1 (1)
Practising status	
Currently practising	109 (96)
Not practising, plan to return	3 (3)
Not practising, no plans to return	2 (2)
Location of work^a^	
Private practice	97 (87)
Public sector	12 (11)
Public and private	2 (2)
Year of graduation, median (IQR)	2017 (2015–2019)
Endorsement to provide scheduled medicines	
Endorsed	2 (2)
Currently training	3 (3)
Not endorsed, interested in becoming endorsed	50 (44)
Not endorsed, no interest	59 (58)
Dermatoscope usage	
Currently use	15 (14)
Don't currently use, interested in using	66 (60)
Don't currently use, not interested in using	30 (27)

Abbreviation: IQR, interquartile range.

^a^ Data missing for *n* = 3.

### 3.2 Preferences for Content and Delivery of Podiatry Programmes

**FIGURE 1:**
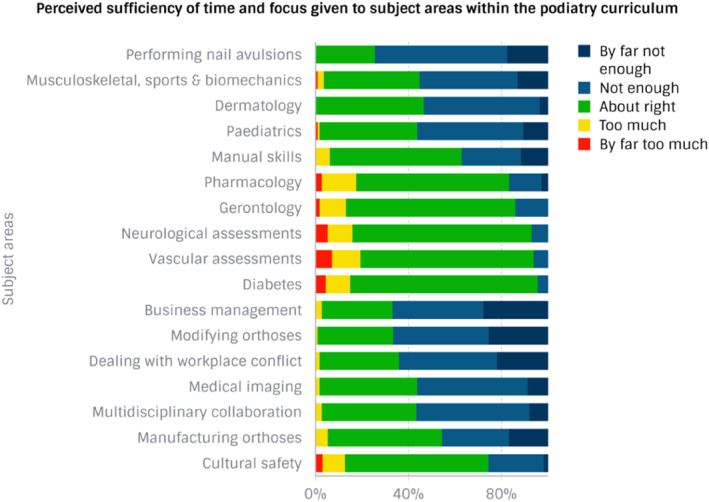
Graduate podiatrists' perceived sufficiency of time and focus given to subject areas within the podiatry curriculum.

Figure 1 presents respondents' perceptions of whether sufficient time and focus were given to subject areas. Respondents were most satisfied with their pre‐registration training in diabetes, neurological and vascular assessments, gerontology, pharmacology and cultural safety. The most commonly reported areas in which respondents felt there was not enough time and focus included performing nail avulsions, business management, modifying orthoses and dealing with workplace conflict. A significantly higher proportion (71%) of private podiatrists compared to public podiatrists (33%) thought there was insufficient time and focus to business management (*p* = 0.02). There was no association between place of work and other topic areas (Figure 1).

**FIGURE 2:**
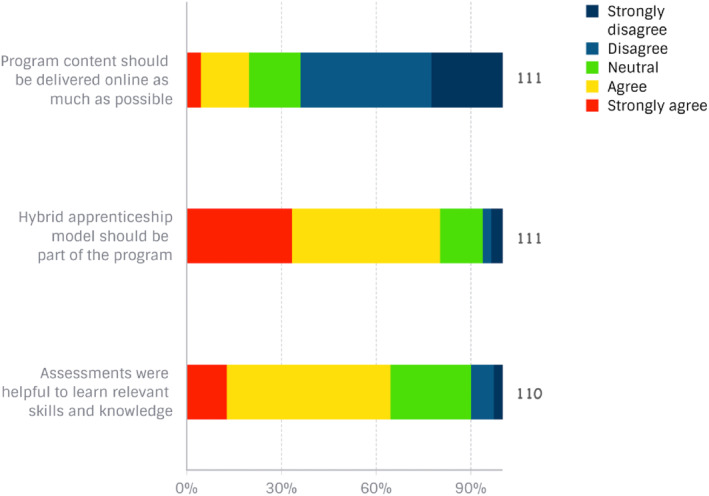
Graduate podiatrists' perceptions on whether the podiatry programme should be delivered online as much as possible, students would benefit from a hybrid‐apprenticeship model and the assessments were relevant to their work as a podiatrist.

**FIGURE 3:**
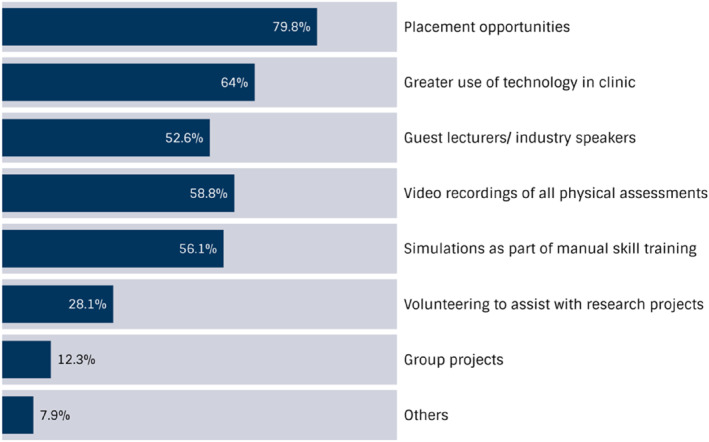
Undergraduate content delivery method preferences of graduate podiatrists.

On whether the podiatry programme should be delivered online as much as possible, most participants disagreed (*n* = 46, 41%) or strongly disagreed (*n* = 25, 23%) (Figure 2). Kendall's tau‐b test found a very weak positive correlation between year graduated and podiatrists' favourable preference for the delivery of the course online (τb = 0.02). On whether students would benefit from a hybrid‐apprenticeship model as part of the training programme, most participants agreed (*n* = 52, 47%) or strongly agreed (*n* = 37, 33%) (Figure 2). Similarly, most participants agreed (*n* = 57, 52%) or strongly agreed (*n* = 14, 13%) that the assessments in podiatry subjects were relevant to their work as a podiatrist (Figure 2). Over half of participants (*n* = 62, 56%) preferred that endorsement for scheduled medicines be embedded within the programme for eligible students only, whereas 27 (24%) respondents preferred all graduates to be endorsed and 22 (20%) preferred this to remain a postgraduate pathway. The following delivery methods were the most highly voted by respondents to have enhanced their learning: placement opportunities (*n* = 91, 79.8%); greater use of technology (*n* = 73, 64%); video recordings of physical assessments (*n* = 67, 58.8%); and simulations within manual skills training (*n* = 64, 56.1%) (Figure 3).

### 3.3 Additional Skills and Technology for Podiatry Training

Four categories emerged from the inductive content analysis of responses regarding additional skills and technology:

1. Introduction of modern technology in the curriculum
2. Greater emphasis on musculoskeletal and biomechanics training
3. More in‐depth training on wound care management
4. Enhanced focus on routine podiatric care

#### 3.3.1 Category 1: Introduction of Modern Technology in the Curriculum

The podiatrists expressed interest in training in use of modern devices such as shockwave therapy, ultrasound, laser and digital scanning for orthotic prescription, with shockwave therapy being the most frequently mentioned.

I wish we had been able to know more about technologies frequently used in practice like laser treatment for warts and fungal nails, shockwave therapy… uni would have provided a safer opportunity for us to practise these skills.

#### 3.3.2 Category 2: Greater Emphasis on Musculoskeletal and Biomechanics Training

Many responses stressed the need for more streamlined musculoskeletal and biomechanics education, with focus on identifying and managing lower limb conditions. Participants also suggested incorporating additional treatment methods, such as dry needling and manual therapies, into the pre‐registration programme.

Every single student that I've hosted on placement has struggled with confidence in clinical decision making and making observations with gait assessments and diagnostics.

#### 3.3.3 Category 3: More In‐Depth Training on Wound Care Management

Many respondents identified a current lack of depth in wound management and high‐risk foot management training in the curriculum. Participants felt that graduates should be equipped with better skills and knowledge in wound debridement and dressing selection through their undergraduate training.

More content on dressings, how to dress wounds. This is learnt in the field but if you're not taught by a podiatrist with years of experience you don't know how to dress wounds properly.

#### 3.3.4 Category 4: Enhanced Focus on Routine Podiatric Care

Participants also suggested prioritising routine podiatry care. These participants felt that mastering the fundamentals of podiatry care should occur before additional technology is incorporated.

I’d like to see future students trained to be able to complete general care adequately, efficiently and professionally before worrying about any additional skills or technology.

### 3.4 Strategies to Reduce Stress From Assessments

Four Categories Were Identified in Participants' Responses Suggesting Strategies to Alleviate Stress From Clinical Assessments:

1. Incorporating mock assessments within the curriculum
2. Reducing the weightage of clinical assessment
3. Reducing the rigidity of the exam structure
4. Improving the uniformity of educators and reducing biases

#### 3.4.1 Category 1: Incorporating Mock Assessments Within the Curriculum

There was support for simulating the conditions of assessment to allow students to familiarise themselves with the testing process such as the format, focus and allocated timing. Feedback given could highlight strengths and weaknesses of the students and enable them to make necessary improvements in preparation for the actual exam.

The more times they are tested throughout the degree without the pressure of marks, the more confident they become.

#### 3.4.2 Category 2: Reducing the Weightage of Clinical Assessment

Podiatrists proposed assessing clinical competency from a series of clinical practices throughout the semester rather than having a high‐weightage stand‐alone exam at the end of the semester.

… it [clinical assessment] should be set over a longer period and based on a term of internal placements not one stand‐alone exam.

#### 3.4.3 Category 3: Reducing the Rigidity of the Exam Structure

Clinical patient‐based exams are structured to mimic independent working where time‐management is crucial. Respondents suggested ways in which stress could be mitigated through changing the exam structure, such as reducing time constraints in the exam. Other podiatrists suggested for the approach of the exam to reflect a real‐life consultation and place less emphasis on the idea that the exam is the ultimate determinant of success.

In the real world a meeting does not require a stopwatch to present or discuss a case.

#### 3.4.4 Category 4: Improving the Uniformity of Educators and Reducing Biases

Podiatrists indicated that they experienced variability in the guidance and instructions provided by clinical educators. There was also uncertainty in the correctness of certain approaches to patient care. Some podiatrists recommended that examiners should be different from their usual clinical educators to minimise potential biases that might occur during the exam.

… A lot of clinical supervisors give different advice and you are never really sure what to expect. Each lecturer and supervisor should be explaining these types of exams the same way.

## 4 Discussion

This survey explores the preferences of podiatrists for the content and delivery methods of a podiatry programme. The study is timely, as podiatry enrolments have been declining in Australia, New Zealand and the United Kingdom [4, 6]. Podiatrists who took part in our survey were most satisfied with their pre‐registration training in diabetes, neurological and vascular assessments, gerontology, pharmacology and cultural safety. The most commonly reported areas in which respondents felt there was not enough time and focus in the pre‐registration curriculum included performing nail avulsions, business management, modifying orthoses and dealing with workplace conflicts. In the current programme, each podiatry student must perform at least one partial nail avulsion under the supervision of a podiatrist following lectures and practical nonclinical sessions in how to perform a partial nail avulsion. In the final year of study, podiatry students develop a business plan following self‐directed online learning, which covers basic business management and basic conflict resolution. That particular course is exclusively online and completed by students at their own pace while they are rotating through external clinical placements. There are no written exams for that particular course. Throughout the current programme, students perform a limited range of orthoses modifications in their campus‐based orthoses labs and perform orthoses modifications as required in clinic and on clinical placements, with experiences varying between students. These areas have been flagged for extensive revision in the redevelopment of the podiatry programme.

There was strong support for a hybrid‐apprenticeship model and limited support for online programme delivery. Respondents also called for more integration of modern technology, musculoskeletal and biomechanics training, wound care management and routine podiatric care. Suggested strategies to reduce assessment‐related stress included incorporating mock assessments, reducing the weightage of clinical assessments, reducing the rigidity of the exam structure and reducing assessor bias.

Podiatrists in our study called for greater emphasis on hands‐on experiences in the pre‐registration curriculum, with many in favour of a hybrid‐apprenticeship model [20]. Clinical placements give students the opportunity to gain work experience, develop their skills and establish professional connections [21]. The dynamic nature of healthcare demands health practitioners to adapt to the changing patient needs, new technology and evolving patient care frameworks [22]. Through these real‐world experiences, students can develop clinical competence, bridge the gap between classroom learning and patient care and gauge their suitability to the job [23]. However, the availability of placements is subject to many challenges, such as shortage of supervisors and resources, the time commitment required of students and supervisors and the willingness of supervisors to allow students to practice hands‐on skills [23, 24]. Our findings highlight a demand for greater practical experience in the pre‐registration curriculum, although more resources and support are required.

Podiatrists in our study were generally not supportive of transitioning all hands‐off (theoretical learning) to online delivery. At the time of the survey, each lecture was delivered in person, streamed live‐online and recorded for students to watch at a later time/day at their convenience, allowing students to select their preferred method of delivery. All practical sessions and clinics were face‐to‐face. The introduction of online learning has helped improve efficiency, cost‐effectiveness and flexibility, as well as allowing students to engage in part time jobs and personal interests while juggling course programmes [25, 26]. However, online learning can present challenges in establishing a personal connection between students and university educators/academics, which is essential for new students to achieve a sense of belonging [25]. Additional drawbacks include lost opportunities such as obtaining references or work opportunities that could arise through connections between students and educators [27]. Online learning can complement content covered in face‐to‐face sessions, and students and educators can enjoy the benefits of blended learning (a combination of face‐to‐face and online learning) when implemented effectively [28]. Telehealth [29] and web‐based simulation training [30] also offer opportunities in course delivery. Taken together, these findings highlight the complexity of online delivery and indicate the importance of ongoing evaluation regarding different delivery modes.

Over two‐thirds of participants reported that there was insufficient time and focus given to business management, in agreement with previous studies [31, 32]. In Australia, a large proportion of podiatrists are sole proprietors [33]. In view of the large percentage of graduates who will likely enter private practice, our findings emphasise the need for greater business management training in pre‐registration curricula. Future research could identify specific aspects of business management needed in the curricula.

We found that pursuing endorsement to prescribe scheduled medicines was met with mixed, but broadly positive, views from practising podiatrists. Nonmedical prescribing following endorsement has the potential to address the increasing demands in healthcare by enhancing efficiency, cost‐effectiveness and accessibility of medicines to patients [34]. According to the podiatry workforce analysis by the Podiatry Board of Australia (2023; 2018), there has been more than a two‐fold increase in the number of podiatrists endorsed for scheduled medicines in the last five years. This suggests that podiatrists are actively seeking to become endorsed although barriers still exist [16, 35]. Although endorsement could largely be a positive step for podiatrists and the broader healthcare system, challenges regarding curriculum expansion and the availability and accessibility of staff and resources need to be considered [35], a recommendation supported by our study.

## 5 Limitations

There are a few limitations to consider. First, we only surveyed podiatrists who were graduates of the University of Newcastle, Australia, podiatry programme and who were members of the Facebook group. Approximately two‐thirds of podiatry graduates from the University of Newcastle belong to the Facebook group and our survey achieved a high response rate. Second, this survey was developed for this study and has not undergone rigorous testing regarding reliability and validity, although we received feedback about the survey from nonacademic staff prior to distribution. Finally, interpretation and generalisability of our findings are limited as only graduates of the University of Newcastle Podiatry Programme were eligible to participate, and we did not collect demographic information about respondents in the interests of anonymity. Nevertheless, we argue that our findings are relevant in countries in which podiatrists are allied health professionals, although our study is less relevant in countries such as the United States where podiatrists are medical specialists.

## 6 Conclusion

In this survey of 114 podiatrists who graduated from the University of Newcastle Podiatry Programme, we found that most were satisfied with their pre‐registration training in diabetes, neurological and vascular assessments, gerontology, pharmacology and cultural safety. The most commonly reported areas in which respondents felt underprepared included nail avulsions, business management, modifying orthoses and dealing with workplace conflicts. There was strong support for a hybrid‐apprenticeship model and our results suggest exercising caution with overreliance on online delivery. Respondents also called for more integration of modern technology, musculoskeletal and biomechanics training, wound care management and routine podiatric care. Suggested strategies to reduce assessment‐related stress included incorporating mock assessments, reducing the weightage of clinical assessments, reducing the rigidity of the exam structure and reducing assessor bias. The results of the study provide a guide to future podiatry curricula at the University of Newcastle, ensuring programme content aligns with contemporary practices, industrial demands and patient needs, as well as producing competent and well‐prepared podiatrists.

## Author Contributions

**Wen Ting Hazel Chua:** conceptualization, data curation, investigation, writing – original draft, writing – review and editing. **Antoni Fellas:** conceptualization, supervision, writing – review and editing. **Andrea Coda:** conceptualization, supervision, writing – review and editing. **Fiona Hawke:** conceptualization, data curation, formal analysis, investigation, methodology, project administration, supervision, writing – review and editing.

## Ethics Statement

The survey and methodology for this study were approved by the Human Research Ethics Committee of the University of Newcastle (Ethics approval number: H‐2023‐0295).

## Conflicts of Interest

The authors declare no conflicts of interest.

## Data Availability

The data that support the findings of this study are available from the corresponding author upon reasonable request. Records will be stored electronically for 5 years post‐publication.
